# Prolactin and Dexamethasone Regulate Second Messenger-Stimulated Cl^−^ Secretion in Mammary Epithelia

**DOI:** 10.1155/2012/192142

**Published:** 2012-07-25

**Authors:** Utchariya Anantamongkol, Mei Ao, Jayashree Sarathy nee Venkatasubramanian, Y. Sangeeta Devi, Nateetip Krishnamra, Mrinalini C. Rao

**Affiliations:** ^1^Department of Physiology and Biophysics, University of Illinois at Chicago, Chicago, IL 60612, USA; ^2^Department of Physiology, Faculty of Science, Mahidol University, Bangkok 10400, Thailand; ^3^Department of Biological Sciences, Benedictine University, Lisle, IL 60532, USA; ^4^Obstetrics, Gynecology and Reproductive Biology, Michigan State University, Grand Rapids, MI 49503, USA

## Abstract

Mammary gland ion transport is essential for lactation and is regulated by prolactin and glucocorticoids. This study delineates the roles of prolactin receptors (PRLR) and long-term prolactin and dexamethasone (P-D)-mediation of [Ca^2+^]_i_ and Cl^−^ transport in HC-11 cells. P-D (24 h) suppressed ATP-induced [Ca^2+^]_i_. This may be due to decreased Ca^2+^ entry since P-D decreased transient receptor potential channel 3 (TRPC3) but not secretory pathway Ca^2+^-ATPase 2 (SPCA2) mRNA. ATP increased Cl^−^ transport, measured by iodide (I^−^) efflux, in control and P-D-treated cells. P-D enhanced I^−^ efflux response to cAMP secretagogues without altering Cl^−^ channels or NKCC cotransporter expression. HC-11 cells contain only the long form of PRLR (PRLR-L). Since the short isoform, PRLR-S, is mammopoietic, we determined if transfecting PRLR-S (rs) altered PRLR-L-mediated Ca^2+^ and Cl^−^ transport. Untreated rs cells showed an attenuated [Ca^2+^]_i_ response to ATP with no further response to P-D, in contrast to vector-transfected (vtc) controls. P-D inhibited TRPC3 in rs and vtc cells but increased SPCA2 only in rs cells. As in wild-type, cAMP-stimulated Cl^−^ transport, in P-D-treated vtc and rs cells. In summary, 24 h P-D acts via PRLR-L to attenuate ATP-induced [Ca^2+^]_i_ and increase cAMP-activated Cl^−^ transport. PRLR-S fine-tunes these responses underscoring its mammopoietic action.

## 1. Introduction

Prolactin is critical for the development of the mammary gland into a secretory type gland during lactation. Either acting alone or in concert with other hormones, prolactin has a plethora of effects on mammary epithelial function during lactation. Amongst other functions prolactin stimulates the production and/or secretion of casein, lipid [1], amino acids [2], and lactose [3] and activates ion transport processes such as those of sodium (Na^+^), chloride (Cl^−^), iodide (I^−^), and calcium (Ca^2+^) [4–6]. An increase in intracellular Ca^2+^ ([Ca^2+^]_i_) in the mammary epithelium can serve two functions—it can contribute to the increased Ca^2+^ content of milk seen during lactation and it can serve as a signaling molecule to stimulate cell function, including fluid, that is, Cl^−^ secretion, necessary for milk production. Although many studies describe the effect of prolactin on Ca^2+^ or on fluid transport, there are few studies linking these effects to the two roles of Ca^2+^. Furthermore the studies are often performed in different animal or cell model systems making inferences difficult. The present study attempts to delineate interplay between hormonal mediation of Ca^2+^ transporters and fluid secretion, in a single model system, the nontransformed mouse mammary epithelial cell line, HC-11.

Prolactin exerts its pleiotropic effects by acting via the transmembrane receptor, PRLR, a member of the cytokine receptor superfamily [7]. Alternative splicing of the PRLR gene results in isoforms of varying lengths [8]. Most prominent are the long (PRLR-L) and short (PRLR-S) isoforms whose expression is both species and organ specific [9, 10]. They may also differ in their C-terminal sequences as seen in the mouse receptors where PRLR-S has a a stretch of 23 aminoacids not seen in PRLR-L. The downstream signaling mechanisms associated with the long form of PRLR have been well studied and implicate many kinases including Janus-, Src-, MAP- and Phosphoinositide 3-kinases [7]. While, not much is known about how prolactin acts via the PRLR-S, it is clear that it is a pathway distinct from that used by the long form of PRLR [11, 12]. Recent studies demonstrate that prolactin may be utilizing the complementary functions of the two isoforms to elicit its final biological effect. For example, PRLR-L alone is not sufficient to maintain progesterone production and fertility despite the activation of Jak2/STAT5 signaling and both PRLR-L and PRLR-S are required for normal female fertility [13]. Secretion of nutrients and electrolytes to form milk involves transcellular and paracellular mechanisms. Movement of glucose, water, and ions such as Na^+^ and Cl^−^ occur transcellularly across the apical and basolateral membranes resulting in a large gradient for Na^+^, K^+^, and Cl^−^ between the plasma and milk and promoting paracellular movement of water. Further, Ca^2+^, lactose, casein and whey proteins are transported from the Golgi apparatus and secreted into the lumen of the mammary glands via exocytosis.

A picture of the molecular mechanisms underlying transepithelial Ca^2+^ transport to increase the Ca^2+^ content of milk during lactation is beginning to emerge [14]. The current view, based on localization and functional data, is that Ca^2+^ is transported from plasma into mammary epithelial cells via Ca^2+^ channels of the transient receptor potential ion channel (TRP) family. The mRNA and protein of various isoforms of the classical TRP (TRPC) were found in the human mammary cancerous cell lines, MCF-7 and MDA-MB-231 [14]. In rat mammary gland, mRNA expression of TRPC 1, 3, 5, and 6 is increased during lactation [14]. Based on inhibitor studies, it is proposed that either TRPC1 and/or TRPC6 may be responsible for the Ca^2+^-sensitive current triggered by activation of the Ca^2+^-sensing receptor [15, 16]. The exit of Ca^2+^ via the apical membrane was initially thought to occur solely via vesicular exocytosis via casein bound Ca^2+^. Secretory-pathway Ca^2+^-ATPases (SPCAs) localized to the Golgi membrane sequester Ca^2+^ for this exocytotic route [15, 16]. More recently, apical plasma membrane Ca^2+^-ATPases (PMCAs), specifically PMCA2, are suggested to extrude Ca^2+^ into the lumen although the underlying mechanisms in view of low [Ca^2+^]_i_ remain to be elucidated [2]. Reinhardt and colleagues demonstrated that there is a 60% reduction in milk [Ca^2+^] [17], and a modest 6–8 fold increase in SPCA1 expression in mice deficient in PMCA2 [16]. Both PMCA and SPCA protein expression are increased during lactation in rat mammary glands [15, 16]. In addition the Ca^2+^ sensitive receptor appears to regulate PMCA2 expression [18]. In contrast, PMCA2 is not detected in the human MCF-7 cells and prolactin promotes sequestration by increasing SPCA2 mRNA expression, and thereby suppresses ATP-induced increases in [Ca^2+^]_i_ [4].

The second function of Ca^2+^ as a signaling molecule regulating ion transport has been less well-studied. In contrast the long-term effects of prolactin and glucorticoids on ion transport processes in tissue explants and in cell lines have been documented. Thus, these hormones have been implicated in the gradual drop of Na^+^ and Cl^−^ concentrations in milk after the onset of parturition due to the closure of tight junctions [19, 20]. Rillema et al. [6, 21] showed that prolactin elevates Na^+^-I^−^ symporter (NIS) protein and increases I^−^ accumulation in cultured mammary tissues of pregnant mice. In HC-11 cells, 48 h of prolactin and cortisol with additional 1–24 h prolactin exposure increases zinc uptake and the expression of its transporter, Zip3 [22]. In many models, including HC-11 cells, the synthetic glucocorticoid, dexamethasone, potentiates the effect of prolactin. For example, in the induction of casein production in HC-11 and in 31EG4 cells [23] and in tight junction formation in HC-11 cells and in rabbit mammary glands [19, 20].

The secretion of fluid by the mammary epithelium is important in milk production and as in other secretory epithelia, it is most likely dependent on transepithelial ion transport. It has been well established that lactating mammary epithelia contain a functional Na^+^/K^+^ pump in the basolateral membrane. In addition mammary epithelia possess a furosemide-sensitive Na^+^-K^+^-2Cl^−^ cotransporter (NKCC). Thus mammary epithelia possess the necessary machinery—Na^+^/K^+^ pump, NKCC, and Cl^−^ channels for Cl^−^ secretion. In terms of hormonal regulation, we previously showed that 10 min exposure to prolactin activated Cl^−^ transport through the phosphorylation of JAK2/STAT5 in HC-11 cells. This in turn increases phosphorylation of NKCC-1, the transporter responsible for Cl^−^ entry into the cell [24]. The HC-11 cells also possess channels needed for Cl^−^ exit, namely, the cystic fibrosis transmembrane conductance regulator (CFTR) and Ca^2+^-dependent Cl^−^ channels (ClCa) [25, 26]. Though our microarray study in pregnant rats showed that lactation induced a transient increase in the expression of chloride intracellular channel 6 (Clic6) [27], these studies did not examine function. However, the effects of long term exposure to prolactin on Cl^−^ transport are not known.

Since the prolactin receptor has multiple isoforms, it is conceivable that it elicits its effects on ion transport via different receptors. For example, the mouse mammary gland possesses one long and three short isoforms of PRLR [9]. Mice with the homozygous PRLR knockout become sterile and therefore cannot be used to study mammary development [28]. The heterozygous PRLR knockout mice (PRLR±) are fertile but do not exhibit lobulo-alveolar development and milk secretion in young females and fail to lactate after the first pregnancy [29]. Since PRLR-S lacks the cytoplasmic regulatory domain, it was postulated that PRLR-L was responsible for PRL signaling and that PRLR-S was a dominant negative of PRLR-L. However, studies from one of us (Y. S. Devi) and colleagues have demonstrated that mice expressing PRL-RS showed early follicular recruitment and premature ovarian failure [12], and overexpression of short-form PRLR (PRLR-S) into PRLR± mice rescued mammopoiesis and functional development of the mammary gland [30]. The expression of PRLR-S in HC-11 cells is controversial; while Wu et al. [31] reported its presence, we were able to detect only PRLR-L and not PRLR-S in our earlier studies in HC-11 cells [24].

Therefore, in the present study, we aimed to elucidate the long-term effects of prolactin treatment, via PRLR-L, on intracellular Ca^2+^ and Cl^−^ transport in HC-11 cells. By transfecting HC-11 with PRLR-S we further examined if coexpression of both PRLR-L and PRLR-S isoforms altered the response to prolactin.

## 2. Materials and Methods

### 2.1. Reagents

Ovine prolactin was obtained from Dr. Arieh Gertler, the Faculty of Agricultural, Food and Environmental Quality Sciences, the Hebrew University of Jerusalem, Rehovot, Israel. Fluo-3/AM (molecular probes), lipofectamine 2000 transfection reagent and SuperScript II Reverse Transcriptase were from Invitrogen, Carlsbad, CA, USA. RNAeasy Mini Kit was purchased from Qiagen, Valencia, CA, USA. Glass bottom dishes were obtained from MatTek Corporation, Ashland, MA, USA. RPMI1640 containing 1% Nutridoma-SP serum-free media supplement was from Roche Applied Science, Indianapolis, IN, USA. SYBR Green PCR Master Mix was purchased from Applied Biosystems, Carlsbad, CA, USA. GenEluteTM High Perfomance Plasmid Maxiprep Kit was from Sigma-Aldrich, St. Louis, MO, USA. All other reagents were obtained from Sigma-Aldrich or Fischer Scientific, Hannover Park, IL, USA and were of analytical grade.

### 2.2. Cell Culture

The HC-11 cells were grown in RPMI1640 containing 5 *μ*g/mL insulin, 10 ng/mL EGF, and 10% fetalbovine serum. The medium was changed every two days. Cells were plated in 10-cm^2^ dish for RNA preparation, 4-cm^2^ dish for iodide efflux assay, and 2 cm^2^ glass bottom dish for [Ca^2+^]_i_ measurement. During hormone treatment, the medium was changed to RPMI1640 containing 1% Nutridoma-SP serum-free media supplement. Cells were treated with 1 *μ*g/mL dexamethasone for 24 h for dexamethasone-treated cells, 1 *μ*g/mL prolactin for 24 h for prolactin-treated cells, and 1 *μ*g/mL dexamethasone for 24 h, washed, followed by exposure to 1 *μ*g/mL prolactin for another 24 h for prolactin + dexamethasone-treated cells.

### 2.3. PRLR-S Transfection

Expression plasmid for rat PRLR-S [32] was prepared using GenEluteTM High Perfomance Plasmid Maxiprep Kit. After cells reached 70% confluency, PRLR-S plasmid was transfected into cells for 4.5 h using Lipofectamine 2000 transfection reagent and washed with PBS. Cells were subsequently treated with or without prolactin (1 *μ*g/mL) + dexamethasone (1 *μ*g/mL) before performing Ca^2+^ imaging, iodide efflux assays, or RNA extraction procedures.

### 2.4. [Ca^2+^]_i_ Measurement

Cells were loaded with 5 *μ*M Fluo-3/AM in serum-free RPMI1640 for 30 min and washed twice with Krebs-Ringer-Hepes medium (KRH) containing 120 mM NaCl, 5.4 mM KCl, 0.8 mM MgCl_2_, 1 mM CaCl_2_, 11.1 mM glucose, and 20 mM HEPES (pH 7.4). Ca^2+^ signals were captured using a Zeiss LSM510 confocal laser scanning microscope (New York, NY, USA). An Ar/Kr laser was used to excite the Fluo-3 at 488 nm and emission signals were detected at 515 nm. Imaging for [Ca^2+^]_i_ was conducted with a 40X objective for wild-type cells or 10X objective for transfected cells. The fluorescence intensity obtained from individual cells were normalized as a relative ratio from the background and averaged. On the average 70–80% of wild-type cells in a culture dish respond to ATP with a robust signal. In wild-type cells, 10–15 cells that responded to 100 *μ*M ATP were selected in each dish. Dishes of transfected cells were viewed in low magnification so 60–80 cells could be randomly selected to obtain a larger sampling. This is to avoid biasing our selection of ATP-responsive cells since there is always a certain amount of cell to cell variability in the efficiency of transient transfections. Among these, only cells that showed the changes in the relative fluorescence ratio were used for calculating area under the curve. To compare effectively the data of the various transfected cells, the relative fluorescence in response to ATP in vector transfected controls is set at 100% (Figure 2(b)). Average data was collected from 4–6 dishes of each treatment. The area under curve of individual cells was determined by using the following formula (obtained from http://www.duncanwil.co.uk/areacurv.html): [(*f*_1_ + *f*_2_)/2 × (*t*_2_ − *t*_1_)]−[(*b*_1_ + *b*_2_)/2 × (*t*_2_ − *t*_1_)], where *f* = fluorescent intensity changes at each time point, *t* = time (s), *b* = fluorescent intensity of the baseline.

### 2.5. Iodide Efflux Assay

The iodide efflux assay was performed as we had previously described [33], based on the original method of Venglarik et al. [34]. Briefly, attached HC-11 cells were washed twice with iodide-free buffer (136 mM NaNO_3_, 3 mM KNO_3_, 2 mM Ca(NO_3_)_2_, 11 mM glucose, and 20 mM HEPES, pH 7.4) and incubated with iodide-loading buffer (136 mM NaI, 3 mM KNO_3_, 2 mM Ca(NO_3_)_2_, 11 mM glucose and 20 mM HEPES, pH 7.4) for 1 h at room temperature. Cells were washed 3 times rapidly with iodide-free buffer, and then samples were collected every 1 min in iodide free buffer. Iodide content was measured by an iodide-sensitive electrode (Orion 96-53, Fisher Scientific) and a pH/mV meter. The iodide concentrations were determined according to the calibration curve. Results were expressed as fold increase of cumulative iodide efflux as described previously [33]. Iodide efflux was measured in the presence and absence of the following reagents: a cAMP cocktail to elevate intracellular cAMP (containing 10 *μ*M 8-Br-cAMP, 10 *μ*M forskolin and 10 *μ*M 3-isobutyl-1-methylxanthine (IBMX)); 100 *μ*M ATP; 10 *μ*M diphenylamine-2-carboxylic acid (DPC); or 10 *μ*M furosemide.

### 2.6. RT-PCR and Real-Time PCR

RNA was extracted by RNAeasy Mini Kit and reverse transcribed by SuperScript II Reverse Transcriptase. The expression of PRLR-L and PRLR-S in HC-11 cells were analyzed by RT-PCR as described previously [35]. Total mRNA from whole ovary of normal cycling mouse was used as control to detect PRLR-S.

Realtime PCR was done using SYBR Green PCR Master Mix in an ABI 7900HT using the System Software (Applied Biosystems, USA), 200 nM sense and antisense primers (sequences shown in Table 1), and cDNA equivalent to 0.5 *μ*g RNA. The reactions were performed in triplicate and run as follows: 50°C 2 min, 95°C 10 min, and 45 cycles of 95°C 15 s and 60°C 1 min. Data were analyzed using the Relative Quantification (RQ) Manager software and presented as relative expression to L-19 used as an internal control.

### 2.7. Western Blotting

Vector- or prolactin receptor short form-tranfected HC-11 cells were treated ±1 *μ*g/mL prolactin and dexamethasone for 24 h. Cells were then sonicated (~20–25 sec pulses) in homogenization buffer (HB: 1 mM EDTA, 2 mM MgCl_2_, 5 mM ß-mercaptoethanol, 25 mM Tris-HCl, pH 7.4, 1 mM DTT, and protease inhibitor cocktail). The homogenate was centrifuged at 3,000 xg for 1 min to remove cell debris. The protein concentrations were quantified via the Bradford method (BioRad, Hercules, CA, USA) and the proteins analyzed by Western blotting procedures as described previously [36]. Briefly, equal amounts of protein (15 *μ*g) from each lysate were subjected to 4–12% SDS-PAGE and transferred onto PVDF membrane at 250 mAmps for 1.5 h, in transfer buffer (25 mM Tris, pH 8.1 192 mM glycine, 20% methanol, and 0.1% SDS). The membrane was washed in TBS-T (tris-buffered saline: 50 mM tris-HCl, pH 7.4, 150 mM NaCl, and 0.1% tween-20) 3 × 5 min each and blocked in blotto (5% carnation nonfat dry milk in TBS-T) for 1 h at room temperature. The membrane was then incubated with a rabbit polyclonal anti-CFTR antibody (Santa Cruz, CA, USA; 1 : 1000 dilution in TBS-T containing 1% nonfat milk) overnight at 4°C on a shaker. The blots were next washed 3 × 5 min each in TBS-T and incubated with a horseradish peroxidase-conjugated secondary antibody (Santa Cruz, CA; 1 : 10,000 dilution in TBS-T containing 1% nonfat milk) for 1 h at room temperature. The blots were washed 3 × 5 min in TBS-T and then visualized using a SuperSignal West Pico Chemiluminescent Substrate kit (Pierce, Rockford, IL, USA).

### 2.8. Statistical Analysis

Data were expressed as mean ± standard error of mean (SEM) and compared by using one-way ANOVA and Student's *t*-test. *P* < 0.05 is considered significant for all statistical tests.

## 3. Results

### 3.1. Effect of Prolactin on Ca^2+^ Transport

**Figure 1:**
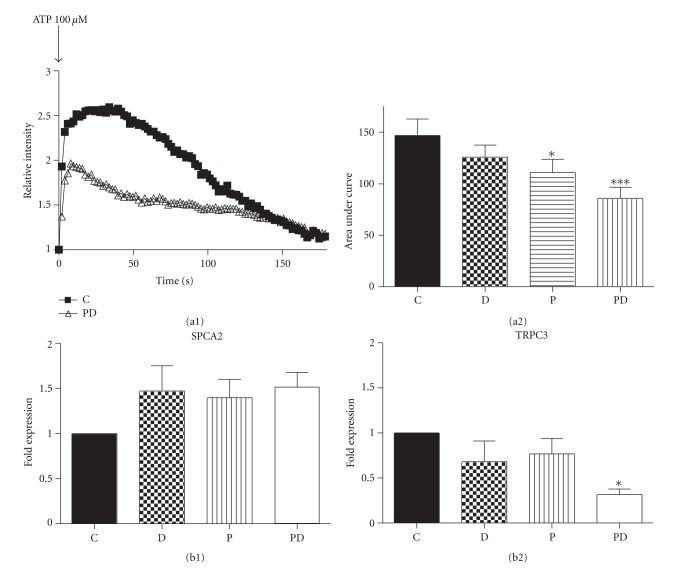
((a1) and (a2)) effect of ATP on [Ca^2+^]_i_ in control and prolactin and/or dexamethasone-treated HC-11 cells. (a1) Representative tracing shows changes of 100 *μ*M ATP-evoked [Ca^2+^]_i_ in control (C) and 24 h 1 *μ*g/mL prolactin + dexamethasone-treated (1 *μ*g/mL) (P-D) cells. [Ca^2+^]_i_ changes were detected from fluorescent intensity of Fluo-3 under confocal microscopy. Data are presented relative to the pretreatment, baseline level. (a2) [Ca^2+^]_i_ changes were calculated as area under curve for (C), prolactin alone (P 1 *μ*g/mL), dexamethasone alone (1 *μ*g/mL) or P-D cells as described in Section 2. Data are mean ± SEM, *n* = 4, where each *n* value represents the mean of 39–44 cells from one dish. ((b1) and (b2)) effect of prolactin and/or dexamethasone treatment on SPCA2 (b1) and TRPC3 (b2) mRNA expression. HC-11 cells were pre-treated with 1 *μ*g/mL prolactin (P) and/or 1 *μ*g/mL dexamethasone (D) for 24 h. Total RNA was extracted, and realtime PCR was performed. L19 was used as internal control. C = control. Data are mean ± SEM, *n* = 3 for SPCA and *n* = 4 for TRPC3. *P < 0.05 and ***P < 0.001 versus control.

We had previously demonstrated that short-term exposure to prolactin did not alter [Ca^2+^]_i_ in HC-11 cells [24] but suppressed ATP-dependent Ca^2+^ increases in MCF-7 cells [4]. The response of MCF-7 cells could be a property of transformed cells. HC-11 cells are a useful in vitro model of mammary cell differentiation; for example, when treated with dexamethasone and prolactin these cells synthesize the milk protein ß-casein. Many studies on milk production and secretion have utilized dexamethasone for its stability and potency. To parallel these models we also utilized dexamethasone in the present study, with the recognition that in the future these results will need to be confirmed with a more nuanced investigation on the efficacy of endogenous glucocorticoids. Therefore we examined if prolactin (1 *μ*g/mL) and dexamethasone (1 *μ*g/mL), either alone or in combination, affects ATP mediated Ca^2+^ release and resequestration in HC-11 cells. Cells were treated with these hormonal regimens for 24 h and changes in [Ca^2+^]_i_ were determined using Fluo-3 and confocal imaging.  Figure 1(a1) shows representative tracings of the changes in [Ca^2+^]_i_ after the addition of 100 *μ*M ATP in control (C) and prolactin + dexamethasone-treated cells. The effects on the magnitude and duration of the Ca^2+^ transient were quantitated by determining the area under the curve as described in Section 2. As shown in Figure 1(a2), prolactin alone or prolactin + dexamethasone decreased the ATP-dependent elevation of [Ca^2+^]_i_ compared to control or cells treated with dexamethasone alone.

The effects of prolactin alone or prolactin + dexamethasone on [Ca^2+^]_i_ could be due to either increased sequestration or decreased entry. Therefore, using mouse-specific primers and real-time PCR, the effects of dexamethasone, prolactin, and prolactin + dexamethasone on the secretory pathway Ca-ATPase, SPCA2 mRNA, an index of altered sequestration, was determined. As shown in Figure 1(b1) prolactin and dexamethasone, either alone or in combination, did not cause any significant change in SPCA2 mRNA expression in HC-11 cells. Therefore we examined if prolactin was attenuating the Ca^2+^ response to ATP by decreasing Ca^2+^ entry via pathways such as the store-operated Ca^2+^ channels, TRPCs. We found that untreated HC-11 cells express TRPC isoforms 1–7, with TRPC3 mRNA exhibiting the highest level of expression (Anantamongkol, Krishnamra, and Rao, data not shown). We next compared the effect of dexamethasone, prolactin, and prolactin + dexamethasone treatment on TRPC3 mRNA expression by real-time PCR. As shown in Figure 1(b2), only prolactin + dexamethasone suppressed TRPC3 mRNA expression, to 30% of the control group. Collectively, these results suggest that prolactin and dexamethasone lower the [Ca^2+^]_i_ response to ATP, by decreasing TRPC3 expression and thereby Ca^2+^ entry. The effects of dexamethasone on enhancing prolactin action are in keeping with published reports and therefore we focused our remaining studies on the actions of prolactin + dexamethasone.

### 3.2. Influence of Prolactin Receptor Isoforms on [Ca^2+^]_i_ Response

**Figure 2:**
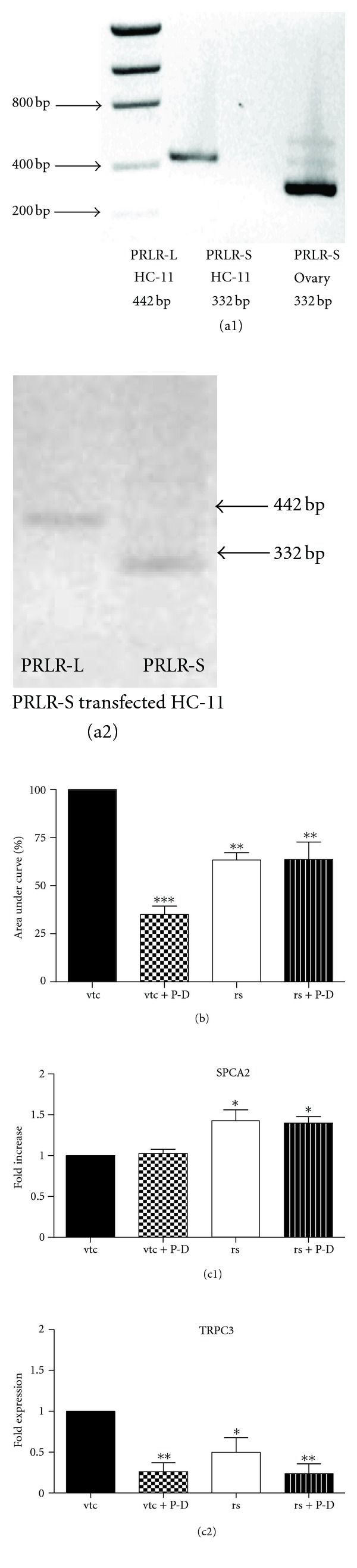
((a1) and (a2)) messenger RNA expression of prolactin receptor long form (PRLR-L) and short form (PRLR-S) in HC-11 and mouse ovary (a1) and PRLR-S-transfected HC-11 cells (a2). (a1) PRLR-L (442 bp), but not PRLR-S (332 bp), is found in HC-11 cells. PRLR-S is present in the ovary cells. (a2) HC-11 cells were transfected with PRLR-S, and both PRLR-L and PRLR-S can be detected in transfected HC-11 cells. ((b) and (c)) vector-transfected HC-11 cells (vtc) or prolactin receptor short form (PRLR-S)-transfected cells (rs) were pretreated with or without 1 *μ*g/mL prolactin and dexamethasone (P-D) for 24 h. (b) Effect of ATP on [Ca^2+^]_i_ in vector-transfected or PRLR-S tranfected cells with or without prolactin and dexamethasone treatment. 100 *μ*M ATP-evoked [Ca^2+^]_i_ changes are calculated as area under curve as described in Section 2. Data are mean ± SEM, and are normalized to vtc, *n* = 4. ((c1) and (c2)) expression of mRNA of SPCA2 (c1) and TRPC3 (c2) in vector-transfected (vtc) or PRLR-S-tranfected (rs) cells ± prolactin and dexamethasone treatment. Total RNA was extracted, and realtime PCR was performed. The mRNA of ribosomal protein L19 was used as an internal control. Data represent mean ± SEM, and are normalized to vtc, *n* = 4. *P < 0.05, **P < 0.01, and ***P < 0.001 versus vtc.

We had reported detecting only the long form of the PRLR in HC-11 cells [24]. However, Wu et al. [31] documented both PRLR-L and PRLR-S in these cells and suggested a role for PRLR-S in modulating casein production. Therefore, we first reevaluated the types of PRLR expressed in our cultures of HC-11 cells. As shown in Figure 2(a1), we were still able to detect only mRNA of the long-form of PRLR (PRLR-L) in HC-11 cells whereas the short-form PRLR-S was detected only in the ovary of the normal cycling mouse. This implies that the above described observations, of prolactin action on ATP-induced [Ca^2+^]_i_ changes are mediated by PRLR-L in HC-11 cells.

To determine if PRLR-S could play a role in the long-term effects of prolactin, HC-11 cells were transiently transfected with PRLR-S. As shown in Figure 2(a2), transfection of PRLR-S (2 *μ*g) into HC-11 cells resulted in the mRNA expression of PRLR-S and these cells also contain PRLR-L (Figure 2(a2)). The changes of [Ca^2+^]_i_ in response to 100 *μ*M ATP in the cells transfected with vector alone (vtc) and the PRLR-S (rs) transfected cells were compared. As in untransfected cells, prolactin + dexamethasone treatment in vector-transfected cells (vtc) showed a greater than 50% reduction in [Ca^2+^]_i_ (Figure 2(b)). PRLR-S transfection, even in the absence of prolactin + dexamethasone showed a 33% reduction in [Ca^2+^]_i_ compared to vector transfected cells. However, these rs cells did not show any further alterations in [Ca^2+^]_i_ in response to ATP after prolactin + dexamethasone treatment (rs+P-D).

As shown in Figure 2(c1), as in the wild-type cells, empty vector transfected cells (vtc) did not show an alteration in SPCA2 mRNA expression. In marked contrast, PRLR-S (rs) transfected cells, either in the presence or absence of 24 h prolactin + dexamethasone treatment showed increases in SPCA2 mRNA expression as compared to control vtc cells (Figure 2(c1)). Transfection of PRLR-S decreased TRPC3 mRNA expression by 50%, and this effect was further suppressed by treatment with prolactin + dexamethasone (Figure 2(c2)). As in the case of wild type cells (compare Figure 2(c2) and Figure 1(b2)), treatment with prolactin + dexamethasone, suppressed TRPC3 expression in vtc cells by about 75%. Thus, transfection of PRLR-S, even in the absence of prolactin + dexamethasone, attenuates the Ca^2+^ signal in response to ATP, presumably by decreasing TRPC3 mRNA and increasing SPCA2 mRNA.

### 3.3. Effect of Prolactin on Cl^−^ Secretion in HC-11 Cells

**Figure 3:**
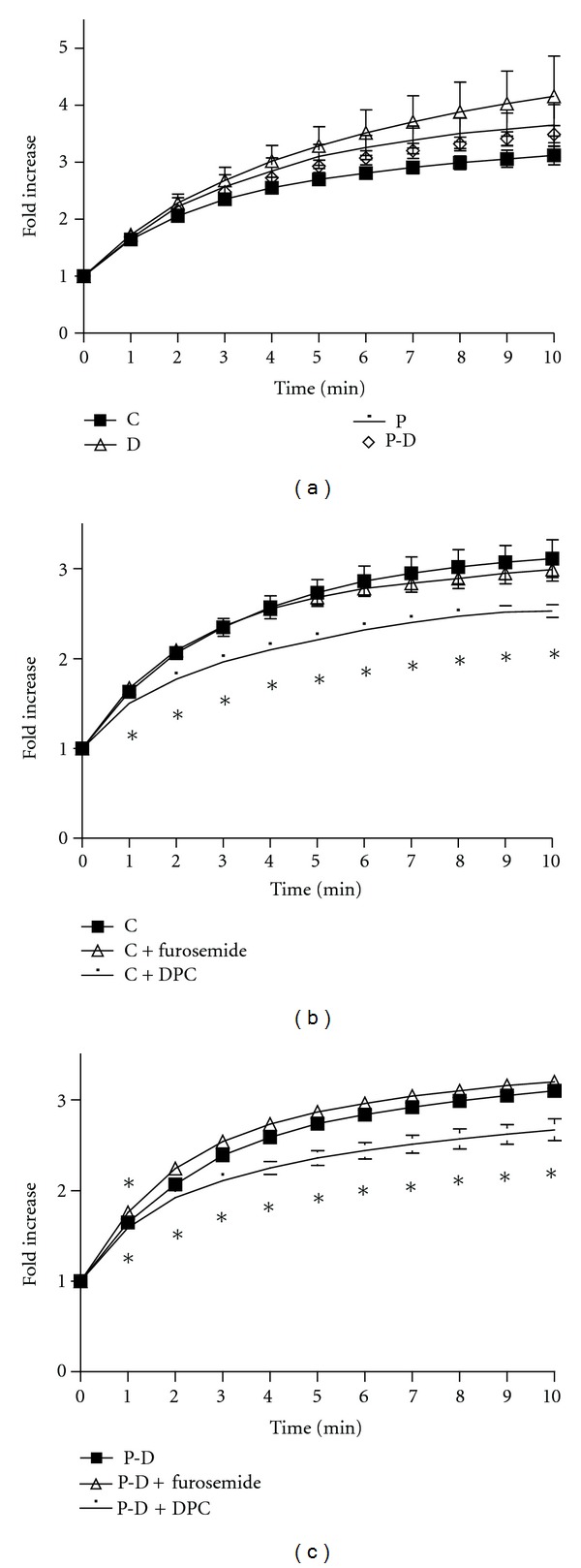
Effect of prolactin and/or dexamethasone (a) DPC and furosemide ((b) and (c)) treatment on cumulative iodide efflux. (a) HC-11 cells were pretreated with 1 *μ*g/mL prolactin (P) and/or 1 *μ*g/mL dexamethasone (D) for 24 h prior to iodide efflux assay ((b) and (c)) effect of 10 *μ*M DPC (a CFTR inhibitor) and 10 *μ*M furosemide (a NKCC1 inhibitor) on iodide efflux in control (b) and 24 h P-D-treated cells (c). Data represent mean ± SEM relative to value at starting point. *n* ≥ 3. *P < 0.05 versus non-inhibitor treated groups.

While short-term (10 min) incubation with prolactin stimulates Cl^−^ transport in HC-11 cells but does not alter [Ca^2+^]_i_ [24], results in Figure 1 suggest that 24 h treatment of these cells with prolactin or prolactin + dexamethasone resulted in a diminution of ATP-induced [Ca^2+^]_i_ elevation. Therefore we probed whether this effect of long-term prolactin action on Ca^2+^ sequestration influences its action on Cl^−^ transport in HC-11 cells. Cl^−^ transport was assessed by the iodide (I^−^) efflux method [34]. As shown in Figure 3, 24 h treatment with prolactin, dexamethasone, and prolactin + dexamethasone did not alter basal I^−^ efflux in HC-11 cells. I^−^ efflux in all these treatments are sensitive to 10 *μ*M DPC, but not to 10 *μ*M of furosemide (as examples, data for control and prolactin + dexamethasone are shown in Figures 3(b) and 3(c), resp.). At these concentrations DPC largely inhibits CFTR and furosemide affects NKCC.

**Figure 4:**
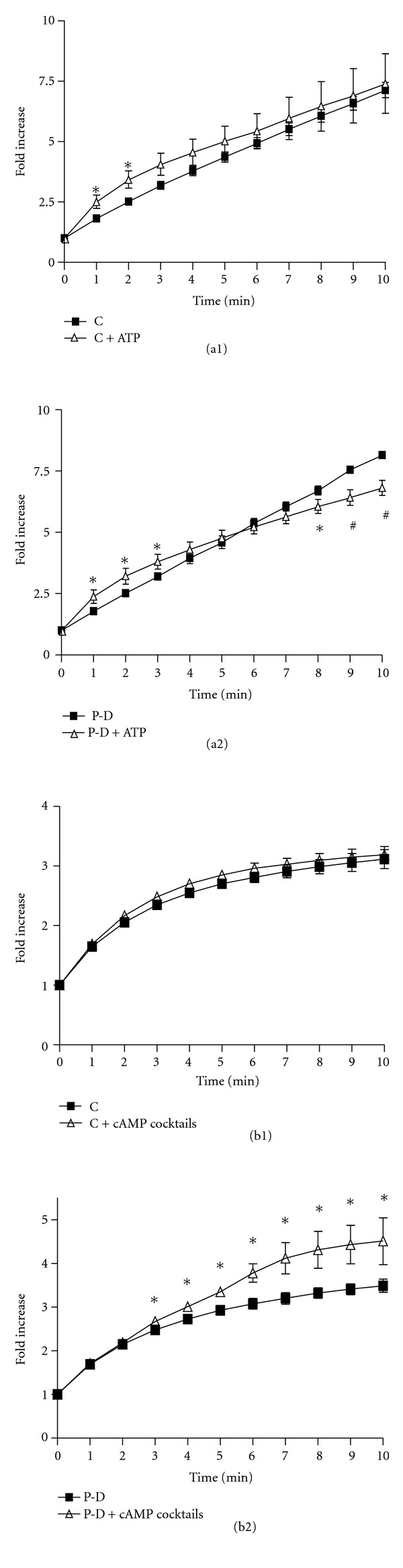
((a1) and (a2)) Effect of ATP on iodide efflux in control (a1) and prolactin and dexamethasone-treated (a2) HC-11 cells. (a1) Iodide efflux assays were performed in the absence or presence of 100 *μ*M ATP. (a2) HC-11 cells were pretreated with 1 *μ*g/mL prolactin and dexamethasone (P-D) for 24 h prior to iodide efflux assay. *n* ≥ 3. *P < 0.05 and ^#^ *P* < 0.01 versus non-ATP treated groups. ((b1) and (b2)) effect of cAMP cocktails on iodide efflux in control (b1) and prolactin and dexamethasone-treated (b2) HC-11 cells. (b1): Iodide efflux assays were performed in the absence or presence of cAMP cocktails (100 *μ*M cAMP, 10 *μ*M forskolin, and100 *μ*M IBMX). (b2) HC-11 cells were pretreated with 1 *μ*g/mL prolactin and dexamethasone (P-D) for 24 h prior to iodide efflux assay. Data represent mean ± SEM relative to value at starting point. *n* ≥ 3. *P < 0.05 versus non-cAMP treated groups.

Next, we examined the effects of the hormone regimen on secretagogue-stimulated Cl^−^ transport. ATP is known to stimulate Cl^−^ secretion in 31EG4 mouse mammary epithelial cells by triggering Ca^2+^ release [37]. In HC-11 cells, ATP (100 *μ*M) caused a rapid and transient (1–3 min) increase in I^−^ efflux of control cells (Figure 4(a1)). Prolactin + dexamethasone-treated cells also show a similar rapid and transient increase in I^−^ efflux at early time point (Figure 4(a2)). However, there was a late decrease in I^−^ efflux in prolactin + dexamethasone-treated cells at 8–10 minutes (Figure 4(a2)). When control, and prolactin + dexamethasone cells were exposed to a cocktail to elevate intracellular cAMP {forskolin to activate adenylyl cyclase, IBMX to inhibit phosphodiesterase and 8-Br-cAMP}, I^−^ efflux was significantly increased only in prolactin + dexamethasone-treated cells (Figures 4(b2) versus 4(b1)).

**Figure 5:**
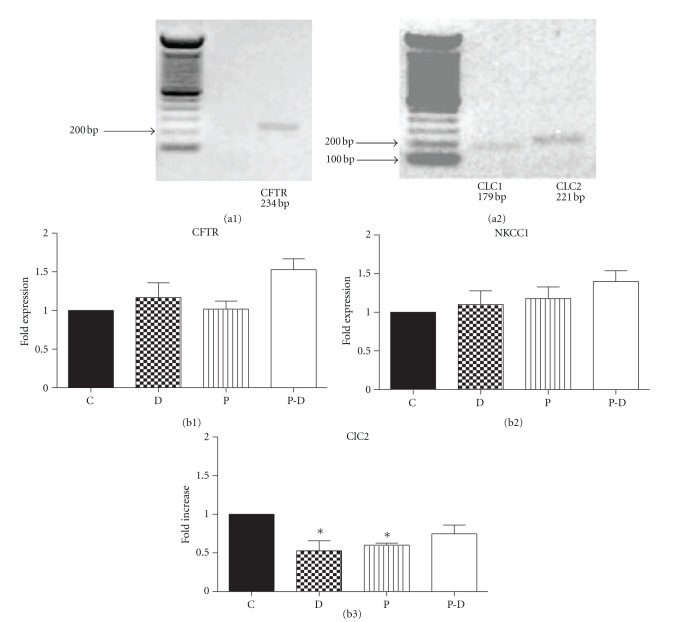
((a1) and (a2)) mRNA expression of CFTR (234 bp) (a1), and ClC1 (179 bp), ClC2 (221 bp) (a2) in HC-11 cells. Representative agarose gel image shows ethidium bromide-stained PCR products. (b1)–(b3) effect of prolactin and/or dexamethasone treatment on CFTR (b1), NKCC1 (b2), and ClC2 (b3) mRNA expression in HC-11 cells. HC-11 cells were treated with or without 1 *μ*g/mL prolactin (P) and/or dexamethasone (D) for 24 h. Total RNA was extracted, and realtime PCR was performed. Ribosomal protein L19 mRNA was used as internal control. C = control. Data represent mean ± SEM relative to control, *n* = 4 for CFTR and NKCC1, and *n* = 3 for ClC2. *P < 0.05  versus control.

To determine if the increase in responsiveness to cAMP was related to the expression of transporters associated with Cl^−^ transport, mRNA expression of key Cl^−^ transporters were assessed by RT-PCR. As previously reported, HC-11 cells contain CFTR (Figure 5(a1)) and NKCC1 [24]. In contrast to the report of Elble et al. [25], ClCa mRNA could not be detected (data not shown). However, we report for the first time that these cells possess ClC-1 and ClC-2 (Figure 5(a2)), members of the ClC family known to be present on the plasma membrane. In addition, ClC-2 is associated with transepithelial Cl^−^ transport [38, 39]. Realtime PCR was used to assess the mRNA expression of these transporters in response to the different hormonal regimens. Twenty-four hour treatment with any of the treatments, prolactin alone, dexamethasone alone or prolactin + dexamethasone, did not alter the mRNA expression of CFTR and NKCC1 (Figures 5(b1) and 5(b2), resp.). Interestingly, in HC-11 cells, prolactin and dexamethasone, suppress the expression of CLC-2 when treated individually while prolactin + dexamethasone did not cause a significant change (Figure 5(b3)).

**Figure 6:**
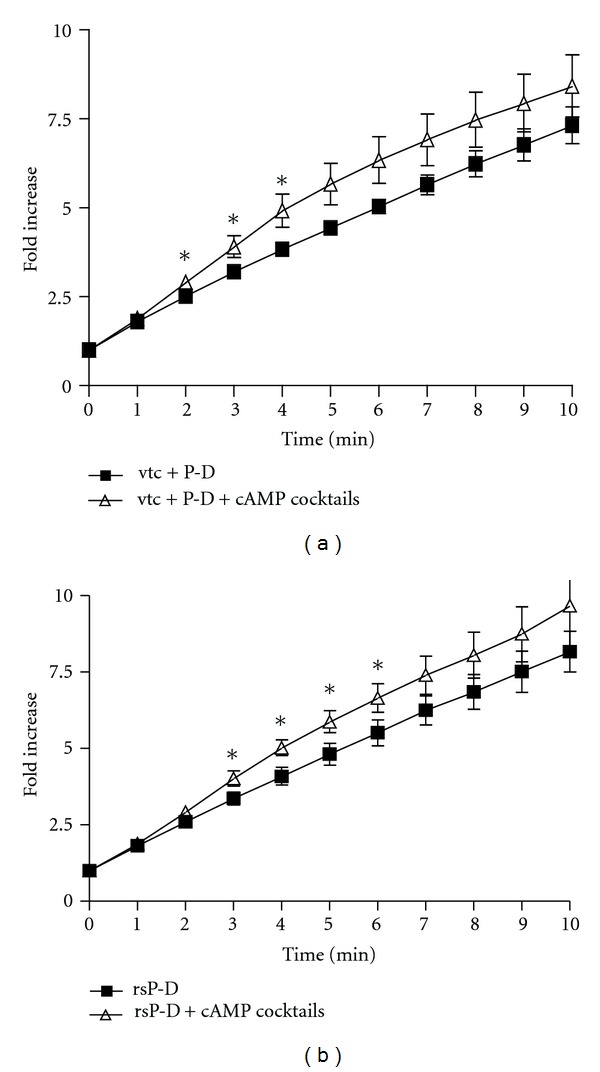
Effect of cAMP cocktail on iodide efflux in prolactin and dexamethasone treated, vector- (a) or PRLR-S-transfected (b) HC-11 cells. Vector transfected HC-11 cells (vtc, (a)) and PRLR-S-transfected cells (rs, (b)) were treated with 1 *μ*g/mL prolactin and dexamethasone (P-D) for 24 h prior to the iodide efflux assays. Iodide efflux assays were performed in the absence or presence of the cAMP cocktail (100 *μ*M cAMP, 10 *μ*M forskolin, 100 *μ*M IBMX). Data represent mean ± SEM relative to value at starting point. *n* ≥ 3. *P < 0.05 versus non-cAMP treated groups.

**Figure 7:**
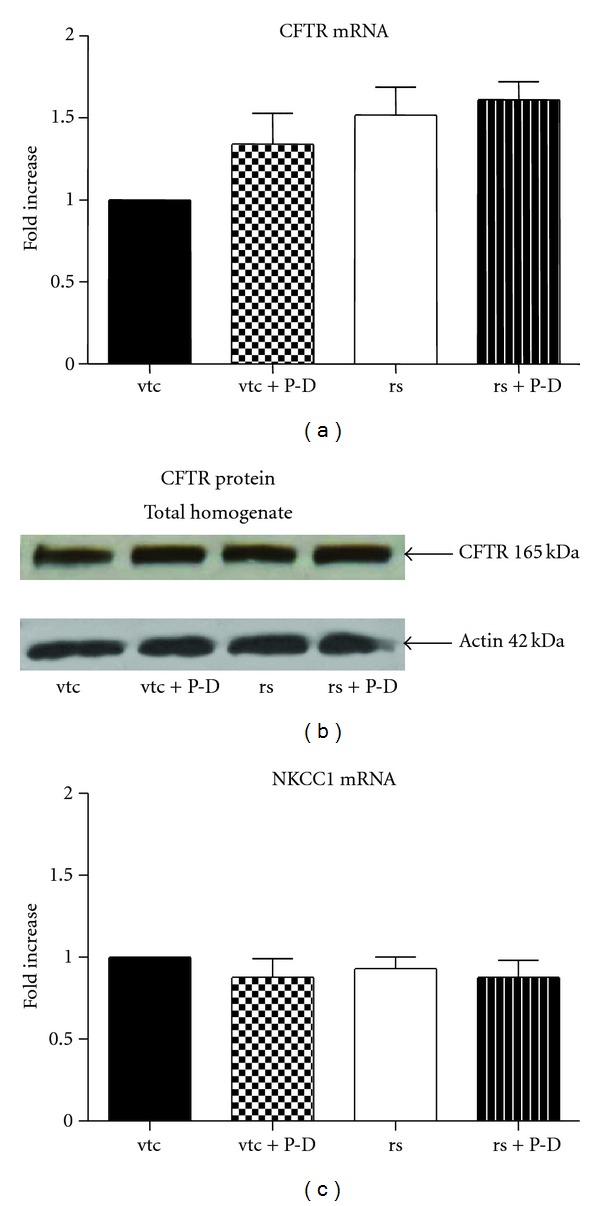
Expression of CFTR mRNA (a), protein (b) and of NKCC1 mRNA (c) in vector-transfected control or PRLR-S-tranfected (rs) HC-11 cells. HC-11 cells were transfected with vector (vtc) or PRLR-S. Transfected cells were treated with or without 1 *μ*g/mL prolactin and dexamethasone (P-D) for 24 h. ((a) and (c)) Total RNA was extracted, and realtime PCR was performed using ribosomal L19 as internal control. (b) Total homogenate of cells were subjected to SDS-PAGE and immunoblotting and probed with a polyclonal CFTR antibody. The blot is representative of 3 experiments. Data represent mean ± SEM relative to vtc, *n* = 3 for CFTR and *n* = 4 for NKCC1.

Both vector-transfected cells (Figure 6(a)) and cells transfected with PRLR-S (rs) (Figure 6(b)), showed an increase in I^−^ efflux in response to the cAMP cocktail. Response in the former was slightly faster than in the latter cells. Neither vector-transfected nor PRLR-S transfected cells, exhibited changes in CFTR or NKCC mRNA expression in the presence or absence of prolactin + dexamethasone-treatment (Figures 7(a) and 7(c)). These results are qualitatively similar to those exhibited by wild type cells (Figures 5(b1) and 5(b2)). Finally Western blotting, confirmed that the protein expression of CFTR was not altered by either PRLR-S transfection or hormonal treatment (Figure 7(b)).

## 4. Discussion

The actions of prolactin on mammary epithelial function are complex and occur via at least two major receptor isoforms and can involve varied signaling pathways. In addition, prolactin's actions can be further modulated by other hormones, specifically glucocorticoids. Dissecting the molecular basis of these actions is compounded by nuances in times of exposure to prolactin, and variability in the models used including species and in the case of cell lines, differences in transformed and nontransformed cells. For example, prolactin is known to alter mammary epithelial Ca^2+^ transport and Cl^−^ transport, and yet there are no systematic studies examining these two actions in the same model system. Equally important, in addition to augmenting milk Ca^2+^ content, an increase in [Ca^2+^]_i_ can serve as a second messenger stimulus of cell function, including Cl^−^ secretion. This regulation clearly is physiologically relevant during lactation. Therefore, this study focused on examining the long-term effect of prolactin on Ca^2+^ responsiveness and Cl^−^ transport in the normal mouse mammary epithelial cell line, HC-11.

The HC-11 cell line was selected as it offered some useful features. The HC-11 cells available to us contain only the long form of PRLR ([24] and Figure 2(a1)). Second, against this backdrop these cells serve as a good model in which to transfect and examine the effects of short form-PRLR. Finally, this cell line has previously been characterized with respect to Cl^−^ transport [24]. Therefore studies were conducted both in nontransfected HC-11 cells containing only PRLR-L and in cells transfected with PRLR-S and therefore containing both PRLR-L and PRLR-S.

A physiologically relevant tool to examine Ca^2+^ and Cl^−^ signaling is ATP. ATP is released upon mechanical stimulation in mammary epithelial cells [40], most likely with relation to myoepithelial contraction facilitating milk secretion. In many cell types, ATP activates plasma membrane P2Y receptors which stimulate a PLC*γ* cascade to signal Ca^2+^ release from intracellular stores. In another mouse mammary epithelial cell line, 31EG4, ATP increased Cl^−^ secretion was suggested to be Ca^2+^ dependent but the effects of prolactin were not examined [41].

As shown in Figure 1, wild type HC-11 cells, show an increase in [Ca^2+^]_i_ in response to 100 *μ*M ATP. However, this response is attenuated when the cells are exposed to prolactin alone for 24 h or prolactin in the presence of dexamethasone, but not when exposed to dexamethasone alone (Figure 1(a2)). A decrease in [Ca^2+^]_i_ could be due to increased sequestration, decreased entry, or both. HC-11 cells treated with prolactin + dexamethasone but not in those treated with prolactin or dexamethasone alone, showed a significant decrease in the mRNA expression of TRPC3, the Ca^2+^ channel protein (Figure 1(b2)). Prolactin is presumably affecting these changes through the long form of its receptor, PRLR-L, the only form detectable in wild-type HC-11 cells (Figure 2(a1)). None of these treatment regimens had an effect on mRNA expression of SPCA2, the secretory pathway Ca^2+^ ATPase, involved in Ca^2+^ sequestration into cellular compartments. These results differ from the effects of prolonged prolactin exposure in the MCF-7, cancerous human mammary epithelial cell line in two respects. First, in MCF-7 cells, the prolactin-induced decreases in [Ca^2+^]_i_ responses to ATP are linked to an increase in SPCA2 mRNA [4], with no change in TRPC3 mRNA expression (Anantamongkol and Krishnamra, unpublished observations). Second, the effects of prolactin on MCF-7 cells are not enhanced by dexamethasone treatment. It remains to be established if these differences reflect either species or cell line (normal versus cancerous) variations and the functional relationship of the secretory pathway Ca^2+^ ATPases, (SPCAs), the plasma membrane calcium ATPase (PMCAs) and the store operated Ca^2+^ channels (TRPCs) remains to be established. Although not evident in MCF-7 cells, the synergistic actions of prolactin and dexamethasone, has been well documented. For example, in HC-11 cells, both hormones are needed to enhance casein production [23, 32, 42–44], and in the mice, at the end of pregnancy, increases in prolactin and cortisol increase tight junction formation [41].

Fluid secretion across the epithelium requires secretion of ions, in particular, Cl^−^. We had demonstrated earlier that short term (10 minute) incubation of HC-11 cells with prolactin increased Cl^−^ transport via a JAK2/STAT5 pathway involving tyrosine phosphorylation of NKCC1 [24]. This effect was transient and exposure of prolactin up to 1 h did not lead to any further increases in Cl^−^ transport. The present study extended these studies to examining the effects of 24 hr prolactin (± dexamethasone) treatment and of ATP on Cl^−^ transport in HC-11 cells. Chloride transport was assessed using the iodide efflux assay. In all treatment groups (data shown for control and prolactin + dexamethasone, Figures 3(b) and 3(c)), this efflux was diminished by the Cl^−^ channel blocker, DPC, but not by the NKCC cotransporter inhibitor, furosemide. This is not surprising since NKCC is halide-selective and transports only Cl^−^ and Br^−^, but not I^−^ and F^−^ [45], and suggests that I^−^ efflux is occurring via Cl^−^ channels. Similarly, radioactive I^−^ efflux in *Xenopus* oocytes was inhibited by DPC but not by by the NKCC inhibitor bumetanide [46]. As in the case of an 1 h exposure, prolonging the exposure to prolactin to 24 h, did not cause an increase in cumulative I^−^ efflux, (Figure 3(a)); neither did overnight treatment with dexamethasone alone or prolactin + dexamethasone (Figure 3(a)).

As reported for 31EG4 and other cells, ATP acts via a Ca^2+^-signaling system to stimulate Cl^−^ secretion in HC-11 cells. The transient increase in I^−^ efflux (Figure 4(a1)) is characteristic of Ca^2+^-dependent secretagogues in Cl^−^ secretory epithelia [37, 47] and is generally reflective of the transient increase in [Ca^2+^]_i_ induced by ATP (cf.  Figure 1). In prolactin + dexamethasone-treated cells ATP likewise caused a transient early increase in I^−^ efflux (Figure 4(a2)). In addition, there was a late decrease in I^−^ efflux and it is reasonable to postulate that this is due to the suppressing effect of prolactin + dexamethasone on [Ca^2+^]_i_ (Figure 4(a2)). Thus, while prolactin + dexamethasone attenuate ATP-induced [Ca^2+^]_i_ increases, they do not suppress the initial transient stimulation of Cl^−^ transport, a characteristic of ATP-stimulation in secretory epithelia.

Another potent stimulator of Cl^−^ secretion is cAMP and the effects of prolonged prolactin treatment on cAMP-mediated Cl^−^ transport was examined. Addition of a cAMP cocktail to 24 h prolactin + dexamethasone treated cells (Figure 4(b2)), but not to control (Figure 4(b1)) cells or cells treated with prolactin or dexamethasone alone (data not shown), stimulated I^−^ efflux. In addition, long-term incubation with prolactin + dexamethasone did not alter the mRNA expression of Cl^−^ transporters (Figures 5(b1)–5(b3)). Thus, increased Cl^−^ transport seen in the prolactin + dexamethasone-treated cells in response to cAMP cocktail (Figure 4(b2)) are most likely due to alterations in transporter function and not expression.

In the present study both ATP and long-term prolactin + dexamethasone treatment result in activation of Cl^−^ transport as measured by iodide efflux. The three “candidate” routes for this transport are CFTR, Ca^2+^-dependent Cl^−^ channels (ClCa1 and ClCa2), and members of the ClC family (ClC-1 and ClC-2). Although ClCa1 and ClCa2 were previously reported in HC-11 cells [25], we could not detect them by RT-PCR in the present study. While HC-11 cells express ClC-2 mRNA, we posit that it may not be the transporter responsible for I^−^ efflux, for three reasons. It is uncertain whether I^−^ efflux can measure ClC-2 activity as I^−^ has been shown to be a permanent blocker of ClC [48]. Second, the ClC-2 mRNA expression was suppressed by prolactin or dexamethasone treatment alone (Figure 5(b3)). Finally, there is controversy in the literature whether ClC-2 functions as an apical Cl^−^ channel [49], a lateral membrane channel [26, 50], or a basolateral channel [51]. In contrast CFTR localizes at the apical membrane in several epithelia such as airway, intestine, pancreas, and sweat gland [52]. HC-11 cells express CFTR, demonstrate DPC-sensitive halide efflux, and we propose that it is the most likely route for Cl^−^ secretion in these cells. The molecular mechanisms underlying these actions remain to be determined and are clearly posttranscriptional.

All of the above actions of prolactin are through the PRLR-L receptor, since HC-11 cells do not possess PRLR-S. While PRLR-L has been extensively studied [9], the physiological function of PRLR-S is less well characterized and controversial. Both a dominant negative effect and other distinct functions of this receptor have been reported [12, 32, 42–44]. However, it is clear that introduction of PRLR-S in the PRLR ± mice brings a distinct change in the development of mammary alveolar glands and compensates for the haploinsufficiency of PRLR-L [30]. Therefore it is not surprising, that in this study, the presence of both PRLR-L and PRLR-S (rs) elicited some distinct responses from wild-type HC-11 cells, with respect to ATP-induced changes in [Ca^2+^]_i_. First, introduction of PRLR-S (rs cells) appears to attenuate the response to ATP even in the absence of additional prolactin + dexamethasone (Figure 2(b)). Second, addition of prolactin + dexamethasone to rs cells did not cause a further decrease in [Ca^2+^]_i_. Third, the attenuation in rs cells is accompanied both by an increase in SPCA2 expression (Figure 2(c1)) and a decrease in TRPC3 expression (Figure 2(c2)). These effects cannot be due to transfection per se as vector-transfected controls showed responses similar to the nontransfected controls in terms of changes in [Ca^2+^]_i_ (Figure 2(b)), lack of change in SPCA2 (Figure 2(c1)) and a decrease in TRPC (Figure 2(c2)). It remains to be determined if in the rs cells, prolactin acts through PRLR-S to increase SPCA2 and through PRLR-L to decrease TRPC3 or if both receptor isoforms are involved in both effects. Regardless, introducing PRLR-S into the cells appears to trigger pathways similar to those evoked by prolactin + dexamethasone treatment with respect to Ca^2+^-signaling. PRLR-S has been shown to physically associate with signaling molecules in the absence of ligand binding [53, 54]. An intriguing possibility may be that PRLR-S associates with molecules that can modulate [Ca^2+^]_i_ such as chemokine receptor family allowing regulation of Ca^2+^-signaling via this receptor independent of prolactin treatment.

The influences of PRLR-S on Cl^−^ transport are more nuanced. First, transfection with vector alone (Figure 6(a)) or with PRLR-S (Figure 6(b)) showed a smaller response to cAMP cocktails as compared to nontransfected cells (Figures 4(b1) and 4(b2)). Second, the time course of stimulation in vector-transfected and PRLR-S transfected cells are slightly different (Figures 6(a) and 6(b)). It remains to be determined if these are due to transfection per se or the presence of PRLR-S. The interplay of PRLR-L and PRLR-S and their signaling pathways in mammary epithelial function is intriguing. The purpose of PRL signaling via different receptors and transduction pathways could be that one isoform serves as a “complementary” or “braking” mechanism for the action of the other, thereby fine tuning the response. As mentioned in the introduction, this notion is supported by recent studies performed on transgenic mice expressing the short or the long form of PRLR selectively [11–13]. For example, the deleterious effect of PRLR-S in the ovary is prevented by the presence of PRLR-L. Ultimately regardless of the differences in receptor isoform consensus domains and signaling pathways the 3D structure will determine the final function. To our best knowledge, no crystal structure or 3D model has been analyzed for the C-terminal domain of either form of the prolactin receptor.

In summary, these results demonstrate that in HC-11 cells, long term prolactin + dexamethasone, act via PRLR-L to modulate the ability of the cell to respond to ATP and to cAMP-dependent secretagogues. In the former case it is due to a dampening of the Ca^2+^ signaling by decreasing Ca^2+^ entry via TRPC3 channels and in the latter by an increase in Cl^−^ secretion, most likely stimulating CFTR function. This modulation is further fine-tuned by the presence of PRLR-S, which appears to obviate the need for exposure to prolactin + dexamethasone by causing a decrease in [Ca^2+^]_i_, both by increasing sequestration via SPCA2 and decreasing entry via TRPC3. These fine-tuning mechanisms may explain the ability of PRLR-S to rescue mammopoiesis in PRLR± mice [30].

## Figures and Tables

**Table 1:** Primer sequences for studying the gene expressions in HC-11 cells.

Genes	Size (bp)	Forward	Backward
mPRLR-L	442	ATACTGGAGTAGATGGGGCCAGGAGAAATC	CTTCCATGACCAGAGTCACTGTCAGGATCT
mPRLR-S	332	ATACTGGAGTAGATGGGGCCAGGAGAAATC	ATATTTGAGTCTGCTGCTTCAGTAGTCAAG
rPRLR-S	332	ATACTGGAGTAGATGGAGCCAGGAGAGTTC	CTATTTGAGTCTGCAGCTTCAGTAGTCA
SPCA2	215	GAAGCCCTTTCTCAGCATGT	TTTCGTTGGCTGTCAGAGTG
TRPC3	153	TAGCACAACGTGGGCAATAA	GGTCAACTGCTGGAACCATT
CFTR	234	ATCAACGGAATCGTCCTACG	AAATCCCTCCTCCCAAAATG
CLCA	425	ACTCGAAGACACGGCTGTATGAAC	CTGTCAAATGTGACTAATCCAAC
ClC1	179	CGAGCTGATCCTGTGAACAA	AATTCTTCCCTGCCCAAGAT
ClC2	221	TGCCTGTCTTTGTCATTGGA	AGGCAGAATGTGAGCGATCT
NKCC1	199	GGCTGGATCAAGGGTGTATTA	ATCGGGCCCAAAGTTCTCATT
L19	194	AGCGCCTCCAGGCCAAGAAGG	CCAGGCCGCTATGTACAGACACGA

## References

[1] Rillema JA, Etindi RN, Cameron CM (1986). Prolactin actions on casein and lipid biosynthesis in mouse and rabbit mammary gland explants are abolished by p-bromphenacyl bromide and quinacrine, phospholipase A2 inhibitors. *Hormone and Metabolic Research*.

[2] McManaman JL, Neville MC (2003). Mammary physiology and milk secretion. *Advanced Drug Delivery Reviews*.

[3] Flint DJ, Gardner M (1994). Evidence that growth hormone stimulates milk synthesis by direct action on the mammary gland and that prolactin exerts effects on milk secretion by maintenance of mammary deoxyribonucleic acid content and tight junction status. *Endocrinology*.

[4] Anantamongkol U, Takemura H, Suthiphongchai T, Krishnamra N, Horio Y (2007). Regulation of Ca^2+^ mobilization by prolactin in mammary gland cells: possible role of secretory pathway Ca^2+^-ATPase type 2. *Biochemical and Biophysical Research Communications*.

[5] Arturi F, Ferretti E, Presta I (2005). Regulation of iodide uptake and sodium/iodide symporter expression in the MCF-7 human breast cancer cell line. *Journal of Clinical Endocrinology and Metabolism*.

[6] Rillema JA, Collins S, Williams CH (2000). Prolactin stimulation of iodide uptake and incorporation into protein is polyamine-dependent in mouse mammary gland explants (44512). *Experimental Biology and Medicine*.

[7] Bouilly J, Sonigo C, Auffret J, Gibori G, Binart N (2012). Prolactin signaling mechanisms in ovary. *Molecular and Cellular Endocrinology*.

[8] Trott JF, Hovey RC, Koduri S, Vonderhaar BK (2004). Multiple new isoforms of the human prolactin receptor gene. *Advances in Experimental Medicine and Biology*.

[9] Bole-Feysot C, Goffin V, Edery M, Binart N, Kelly PA (1998). Prolactin (PRL) and its receptor: actions, signal transduction pathways and phenotypes observed in PRL receptor knockout mice. *Endocrine Reviews*.

[10] Freeman ME, Kanyicska B, Lerant A, Nagy G (2000). Prolactin: structure, function, and regulation of secretion. *Physiological Reviews*.

[11] Devi YS, Shehu A, Stocco C (2009). Regulation of transcription factors and repression of Sp1 by prolactin signaling through the short isoform of its cognate receptor. *Endocrinology*.

[12] Halperin J, Devi SY, Elizur S (2008). Prolactin signaling through the short form of its receptor represses forkhead transcription factor FOXO3 and its target gene Galt causing a severe ovarian defect. *Molecular Endocrinology*.

[13] Le JA, Wilson HM, Shehu A (2012). Generation of mice expressing only the long form of the prolactin receptor reveals that both isoforms of the receptor are required for normal ovarian function. *Biology of Reproduction*.

[14] Shennan DB (2008). Calcium transport by mammary secretory cells: mechanisms underlying transepithelial movement. *Cellular and Molecular Biology Letters*.

[15] Reinhardt TA, Horst RL (1999). Ca^2+^-ATPases and their expression in the mammary gland of pregnant and lactating rats. *American Journal of Physiology, Cell Physiology*.

[16] Reinhardt TA, Filoteo AG, Penniston JT, Horst RL (2000). Ca^2+^-ATPase protein expression in mammary tissue. *American Journal of Physiology, Cell Physiology*.

[17] Reinhardt TA, Lippolis JD, Shulland GE, Horst RL (2004). Null mutation in the gene encoding plasma membrane Ca^2+^-ATPase isoform 2 impairs calcium transport into milk. *Journal of Biological Chemistry*.

[18] Van Houten JN, Neville MC, Wysolmerski JJ (2007). The calcium-sensing receptor regulates plasma membrane calcium adenosine triphosphatase isoform 2 activity in mammary epithelial cells: a mechanism for calcium-regulated calcium transport into milk. *Endocrinology*.

[19] Stelwagen K, McFadden HA, Demmer J (1999). Prolactin, alone or in combination with glucocorticoids, enhances tight junction formation and expression of the tight junction protein occludin in mammary cells. *Molecular and Cellular Endocrinology*.

[20] Zettl KS, Sjaastad MD, Riskin PM, Parry G, Machen TE, Firestone GL (1992). Glucocorticoid-induced formation of tight junctions in mouse mammary epithelial cells in vitro. *Proceedings of the National Academy of Sciences of the United States of America*.

[21] Rillema JA, Yu TX, Jhiang SM (2000). Effect of prolactin on sodium iodide symporter expression in mouse mammary gland explants. *American Journal of Physiology, Endocrinology and Metabolism*.

[22] Kelleher SL, Lönnerdal B (2005). Zip3 plays a major role in zinc uptake into mammary epithelial cells and is regulated by prolactin. *American Journal of Physiology, Cell Physiology*.

[23] Doppler W, Gorner B, Ball RK (1989). Prolactin and glucocorticoid hormones synergistically induce expression of transfected rat *β*-casein gene promoter constructs in a mammary epithelial cell line. *Proceedings of the National Academy of Sciences of the United States of America*.

[24] Selvaraj NG, Omi E, Gibori G, Rao MC (2000). Janus kinase 2 (JAK2) regulates prolactin-mediated chloride transport in mouse mammary epithelial cells through tyrosine phosphorylation of Na^+^-K^+^-2Cl^−^ cotransporter. *Molecular Endocrinology*.

[25] Elble RC, Pauli BU (2001). Tumor suppression by a proapoptotic calcium-activated chloride channel in
mammary epithelium. *Journal of Biological Chemistry*.

[26] Gyömörey K, Yeger H, Ackerley C, Garami E, Bear CE (2000). Expression of the chloride channel ClC-2 in the murine small intestine epithelium. *American Journal of Physiology, Cell Physiology*.

[27] Anantamongkol U, Charoenphandhu N, Wongdee K (2010). Transcriptome analysis of mammary tissues reveals complex patterns of transporter gene expression during pregnancy and lactation. *Cell Biology International*.

[28] Ormandy CJ, Camus A, Barra J (1997). Null mutation of the prolactin receptor gene produces multiple reproductive defects in the mouse. *Genes and Development*.

[29] Brisken C, Kaur S, Chavarria TE (1999). Prolactin controls mammary gland development via direct and indirect mechanisms. *Developmental Biology*.

[30] Binart N, Imbert-Bolloré P, Baran N, Viglietta C, Kelly PA (2003). A short form of the prolactin (PRL) receptor is able to rescue mammopoiesis in heterozygous PRL receptor mice. *Molecular Endocrinology*.

[31] Wu W, Coss D, Lorenson MY, Kuo CB, Xu X, Walker AM (2003). Different biological effects of unmodified prolactin and a molecular mimic of phosphorylated prolactin involve different signaling pathways. *Biochemistry*.

[32] Perrot-Applanat M, Gualillo O, Pezet A, Vincent V, Edery M, Kelly PA (1997). Dominant negative and cooperative effects of mutant forms of prolactin receptor. *Molecular Endocrinology*.

[33] Boonkaewwan C, Ao M, Toskulkao C, Rao MC (2008). Specific immunomodulatory and secretory activities of stevioside and steviol in intestinal cells. *Journal of Agricultural and Food Chemistry*.

[34] Venglarik CJ, Bridges RJ, Frizzell RA (1990). A simple assay for agonist-regulated Cl and K conductances in salt-secreting epithelial cells. *American Journal of Physiology, Cell Physiology*.

[35] Stocco C, Djiane J, Gibori G (2003). Prostaglandin F_2*α*_ (PGF_2*α*_) and prolactin signaling: PGF_2*α*_-mediated inhibition of prolactin receptor expression in the corpus luteum. *Endocrinology*.

[36] Venkatasubramanian J, Selvaraj N, Carlos M, Skaluba S, Rasenick MM, Rao MC (2001). Differences in Ca^2+^ signaling underlie age-specific effects of secretagogues on colonic Cl^−^ transport. *American Journal of Physiology, Cell Physiology*.

[37] Blaug S, Rymer J, Jalickee S, Miller SS (2003). P2 purinoceptors regulate calcium-activated chloride and fluid transport in 31EG4 mammary epithelia. *American Journal of Physiology, Cell Physiology*.

[38] Jentsch TJ, Neagoe I, Scheel O (2005). CLC chloride channels and transporters. *Current Opinion in Neurobiology*.

[39] Jentsch TJ, Poët M, Fuhrmann JC, Zdebik AA (2005). Physiological functions of CLC Cl^−^ channels gleaned from human genetic disease and mouse models. *Annual Review of Physiology*.

[40] Enomoto K, Furuya K, Yamagishi S, Oka T, Maeno T (1994). The increase in the intracellular Ca^2+^ concentration induced by mechanical stimulation is propagated via release of pyrophosphorylated nucleotides in mammary epithelial cells. *Pflugers Archiv European Journal of Physiology*.

[41] Neville MC, McFadden TB, Forsyth I (2002). Hormonal regulation of mammary differentiation and milk secretion. *Journal of Mammary Gland Biology and Neoplasia*.

[42] Lesueur L, Edery M, Ali S, Paly J, Kelly PA, Djiane J (1991). Comparison of long and short forms of the prolactin receptor on prolactin-induced milk protein gene transcription. *Proceedings of the National Academy of Sciences of the United States of America*.

[43] Berlanga JJ, Garcia-Ruiz JP, Perrot-Applanat M, Kelly PA, Edery M (1997). The short form of the prolactin (PRL) receptor silences PRL induction of the *β*-casein gene promoter. *Molecular Endocrinology*.

[44] Huang K, Ueda E, Chen Y, Walker AM (2008). Paradigm-shifters: phosphorylated prolactin and short prolactin receptors. *Journal of Mammary Gland Biology and Neoplasia*.

[45] Russell JM (2000). Sodium-potassium-chloride cotransport. *Physiological Reviews*.

[46] Chao AC, Katayama Y (1991). Regulation of endogenous chloride conductance in xenopus oocytes. *Biochemical and Biophysical Research Communications*.

[47] Barrett KE, Keely SJ (2000). Chloride secretion by the intestinal epithelium: molecular basis and regulatory aspects. *Annual Review of Physiology*.

[48] Miller C (2006). ClC chloride channels viewed through a transporter lens. *Nature*.

[49] Cuppoletti J, Malinowska DH, Tewari KP (2004). SPI^−^0211 activates T84 cell chloride transport and recombinant human ClC-2 chloride currents. *American Journal of Physiology, Cell Physiology*.

[50] Mohammad-Panah R, Gyomorey K, Rommens J (2001). ClC-2 Contributes to Native Chloride Secretion by a Human Intestinal Cell Line, Caco-2. *Journal of Biological Chemistry*.

[51] Peña-Münzenmayer G, Catálan M, Cornejo I (2005). Basolateral localization of native CIC-2 chloride channels in absorptive intestinal epithelial cells and basolateral sorting encoded by a CBS-2 domain di-leucine motif. *Journal of Cell Science*.

[52] Li C, Naren AP (2010). CFTR chloride channel in the apical compartments: spatiotemporal coupling to its interacting partners. *Integrative Biology*.

[53] Devi YS, Seibold AM, Shehu A (2011). Inhibition of MAPK by prolactin signaling through the short form of its receptor in the ovary and decidua: involvement of a novel phosphatase. *Journal of Biological Chemistry*.

[54] Rachel Duan W, Linzer DI, Gibori G (1996). Cloning and characterization of an ovarian-specific protein that associates with the short form of the prolactin receptor. *Journal of Biological Chemistry*.

